# Prostate Fiducial Marker Placement in Patients on Anticoagulation: Feasibility Prior to Prostate SBRT

**DOI:** 10.3389/fonc.2020.00203

**Published:** 2020-02-27

**Authors:** Michelle Iocolano, Seth Blacksburg, Todd Carpenter, Michael Repka, Susan Carbone, Gizem Demircioglu, Maryann Miccio, Aaron Katz, Jonathan Haas

**Affiliations:** ^1^Department of Radiation Oncology, NYU Winthrop Hospital, Mineola, NY, United States; ^2^Stony Brook University School of Medicine, Stony Brook, NY, United States; ^3^Department of Urology, NYU Winthrop Hospital, Mineola, NY, United States

**Keywords:** fiducial markers, radiotherapy, image-guided, anticoagulants, prostate cancer, SBRT

## Abstract

**Background and Purpose:** Fiducial marker placement is required in patients undergoing robotic-based Stereotactic Body Radiotherapy (SBRT) or image-guided radiation therapy (IGRT) for prostate cancer. Many patients take antiplatelet or anticoagulant medication due to other medical comorbidities. They are often required to temporarily discontinue these medications prior to invasive medical procedures as they are prone to bleed. Some patients are unable to discontinue therapy due to an elevated risk of thromboembolic events. The purpose of this study is to report this institution's experience placing fiducial markers in prostate cancer patients who are on chronic antiplatelet or anticoagulant medication.

**Materials and Methods:** From August 2015–March 2019 57 patients on chronic antiplatelet or anticoagulation therapy who were not cleared to stop these medications underwent transrectal ultrasound guided (TRUS) fiducial marker placement for SBRT/IGRT. All patients were monitored by a registered nurse during the procedure for prolonged bleeding that required staff to hold pressure to the area with a 4 × 4 gauze until it resolved. All patients were also called the following day to assess for ongoing bleeding events. Treatment planning CT scan confirmed the ideal geometry of the marker placement.

**Results:** All 57 patients on antiplatelet or anticoagulant medication who underwent fiducial marker placement were discharged home the same day of the procedure. Four patients experienced persistent bleeding that required a nurse to hold prolonged pressure to the area. No patient experienced significant bleeding the following day or any untoward cardiovascular event.

**Conclusions:** This series suggests the use of antiplatelet or anticoagulant medication is not an absolute contraindication to fiducial marker placement in patients undergoing SBRT or IGRT for prostate cancer. These patients should be closely monitored after the procedure for bleeding complications. Practitioners may consider the patient's medical comorbidities, risk factors for thromboembolism, and overall functional status as there is no standardized protocol for discontinuing anticoagulant or antiplatelet therapy for fiducial marker placement.

## Introduction

In 2019, it is anticipated that prostate cancer will account for almost 20 percent of new cancer diagnoses in men (1). There are many options for treating localized prostate cancer. Stereotactic body radiation therapy (SBRT) allows precise delivery of a large dose of radiation to the prostate in one to five fractions with toxicity rates comparable to conventionally fractionated radiation (2–5). Placement of fiducial markers prior to radiotherapy is necessary for robotic-based SBRT to account for the six degrees of prostate motion during treatment and to prevent over- irradiation of normal surrounding tissue (6, 7). Fiducials are utilized in the majority of those treated with SBRT delivered with gantry-mounted linear accelerators although their role in platforms equipped with cone beam CT imaging is actively being debated (8).

Many patients with prostate cancer eligible for SBRT are on chronic antiplatelet or anticoagulant medication for other medical comorbidities. Fiducial marker placement is an invasive procedure that confers an elevated risk for bleeding complications in these patients and several institutions require the temporary discontinuation of their medication prior to the procedure (6, 7, 9). For many patients, however, this is not feasible due an elevated risk of thromboembolic events. One potential alternative for patients who require active anticoagulation being treated on a unit with cone beam CT are prostatic calculi as they are prevalent in 85% of patients (10). To our knowledge, no study has assessed the feasibility of maintaining antiplatelet or anticoagulant medication prior to fiducial marker placement. The purpose of this study is to report a high-volume academic institution's experience placing fiducial markers for prostate SBRT in this patient population.

## Materials and Methods

### Patient Population

This study was approved by the institutional review board at NYU Winthrop Hospital. From August 2015-March 2019, 57 patients with prostate cancer scheduled for fiducial marker placement and subsequent SBRT or image guided radiation therapy (IGRT) at NYU Winthrop Hospital were identified and consented to the study. Eligible patients included those on chronic antiplatelet or anticoagulant medication who were not cleared to stop their medication prior to fiducial marker placement. Antiplatelet medications included aspirin and clopidogrel. Anticoagulants included warfarin, heparin, apixaban, rivaroxaban, and dabigatran. There was no exclusion criteria and no patients declined to undergo fiducial marker placement. Fiducials were placed by three radiation oncologists. Nursing staff monitored all patients during the procedure to assess for prolonged bleeding from the perineum which required the use of sustained pressure to the area with a 4 × 4 gauze until it resolved. All patients were also called 1 day after the procedure to assess for persistent bleeding events.

### Treatment Technique

Study participants had four gold fiducial markers placed in the prostate transperineally with transrectal ultrasound guidance prior to SBRT/IGRT. Prior to marker placement, lidocaine gel, and EMLA cream were used to numb the perineum and rectum. Two needles were double loaded with two gold fiducial markers each with a spacer in between. The needles were inserted transperineally into the prostate. Two fiducial seeds were placed at the right and left base and two seeds were placed in the right and left apex. Following withdrawal of the ultrasound transducer and needle, nursing staff provided gentle pressure to the area and monitored patients for persistent bleeding. A treatment planning CT scan was used to confirm the ideal geometry of the marker placement. Patients were planned and treated as previously described (11).

## Results

The average age of the study population was 70.3 with a range of 54–83. All 57 patients who underwent fiducial marker placement were on antiplatelets or anticoagulation and discharged home on the same day of the procedure. Baseline patient characteristics are found in Table 1. As seen in Table 2, most patients were on a single antiplatelet agent (63.2%), followed by a single anticoagulant (17.5%), dual antiplatelet agents (17.5%), and triple anticoagulant/antiplatelet agents (1.8%) at the time of procedure. Medical comorbidities requiring the use of anticoagulation or antiplatelet medication are listed in Table 3. Coronary artery disease (56.1%) and primary prevention (17.5%) were most commonly reported.

**Table 1:** Baseline patient characteristics.

	***n* =**	**(%)**
**Clinical stage**		
Tx	3	5.3
T1c	43	75.4
T2a	2	3.5
T2b	5	8.8
T2c	1	1.8
T3a	3	5.3
T3b	0	0
**PSA**	Mean 9.08 ng/ml (3.0–48.0)	
<10 ng/ml	39	68.4
10–20 ng/ml	16	28.1
>20 ng/ml	2	3.5
**Gleason score**		
6 (3+3)	10	17.5
7 (3+4)	21	36.8
7 (4+3)	14	24.6
8–10	12	21.1
**NCCN risk stratification**		
Low	7	12.3
Intermediate	36	63.2
High	14	24.6

**Table 2:** Antiplatelet and anticoagulant medication.

	***n* =**	**(%)**
**Single agent**		
Antiplatelet		
Aspirin	28	49.1
Clopidogrel	8	14.0
Total	36	63.2
Anticoagulant		
Warfarin	3	5.3
Heparin	3	5.3
Apixaban	1	1.8
Rivaroxaban	2	13.5
Dabigatran	1	1.8
Total	10	17.5
**Dual agent**		
Aspirin + Plavix	6	1.5
Apixaban + Plavix	4	7.0
Total	10	17.5
**Three agents**		
Aspirin + Apixaban + Clopidogrel	1	1.8
Total	1	1.8

**Table 3:** Patient comorbidities requiring use of anticoagulant or antiplatelets.

	***n* =**	**(%)**
Pulmonary embolism	2	3.5
Coronary artery disease	32	56.1
Atrial Fibrillation	3	5.3
Cerebral vascular accident	2	3.5
Primary prevention	10	17.5
Other	3	8.8
Unknown	5	5.3

Four patients experienced transient CTCAE grade 1 bleeding at the time of fiducial placement requiring a registered nurse to provide sustained pressure on the perineum with a 4 × 4 gauze. Two of these patients took Aspirin 81 mg daily; one took Aspirin 162 mg daily; and one patient took Aspirin 81 mg, Eliquis 5 mg, and Plavix 75 mg daily. The following day, these patients reported via telephone they did not experience further bleeding events. No other significant bleeding or cardiovascular events in the peri-procedural window were reported. There were no cases of fiducial remediation in this cohort and all patients subsequently received definitive SBRT for their prostate malignancy without untoward medical event precluding completion of their prostate cancer treatment. No patient had signs or symptoms to indicate further bleeding on initiation of radiation treatment.

## Discussion

SBRT offers patients with low- to intermediate risk prostate cancer the convenience of a shortened treatment course with excellent local control rates and treatment toxicities comparable to other definitive radiation modalities. Patients who opt for fiducial marker placement and subsequent SBRT may also be on antiplatelet or anticoagulant regimens for other medical conditions. A review of the literature suggests patients may be required to temporarily discontinue their medication prior to fiducial placement (6, 7, 9, 12). For some patients this is not possible due to an elevated risk of thromboembolism. There is limited evidence to guide management in these patients with a theoretically increased risk of bleeding following fiducial marker placement.

At our institution, 57 patients on chronic antiplatelet or anticoagulant medication were not found to have significant bleeding events following the placement of fiducial markers for prostate SBRT. While four patients experienced bleeding at the time of procedure, it had resolved with no further complications as per patient report the following day. Similarly, Ihezue et al. suggest that patients on warfarin did not experience clinically significant hematuria, hematospermia or rectal bleeding within 10 days following transrectal ultrasound guided prostate biopsy when compared to those not on anticoagulants (13). While Saito et al. analogously found comparable bleeding risk in patients on anticoagulant or antiplatelet therapy and those who were not, patients on these medications were more likely to have clot retention and minor bleeding following biopsy. Notably, there was no report of significant bleeding events in any patient undergoing transrectal ultrasound guided biopsy (13, 14).

While this is the first study to suggest that chronic antiplatelet or anticoagulant medication is not an absolute contraindication for seed placement, further research is required. The small sample size in this study may be insufficient to detect bleeding complications in the larger patient population eligible for this procedure and a prospective, randomized study with greater patient numbers could provide greater clarity on this topic. While there is no standardized protocol for discontinuing these medications in preparation for fiducial marker placement, practitioners must consider many clinical factors including overall risk of thromboembolism, functional status, other comorbidities prior to making a treatment decision.

## Data Availability Statement

All datasets generated for this study are included in the article/supplementary material.

## Ethics Statement

The studies involving human participants were reviewed and approved by NYU Winthrop Hospital IRB. The patients/participants provided their written informed consent to participate in this study.

## Author Contributions

MI and JH were responsible for the conception of this study. All authors contributed to the writing of this review, editing, and final approval prior to submission.

### Conflict of Interest

The authors declare that the research was conducted in the absence of any commercial or financial relationships that could be construed as a potential conflict of interest.
